# Simultaneous multiple carpometacarpal joint dislocations with trapezium fracture and trapezoid dislocation: A case report

**DOI:** 10.1016/j.tcr.2026.101355

**Published:** 2026-04-27

**Authors:** Yuhei Mogi, Kinya Nishida, Maeda Akane, Uesugi Kazuhiro, Irifune Hideto, Che Yong Ho, Takahiro Iida

**Affiliations:** a Department of Orthopaedic Surgery, Teine Keijinkai Hospital, Sapporo, Japan

**Keywords:** Carpometacarpal joint dislocation, Trapezium fracture, Trapezoid dislocation, Scaphotrapeziotrapezoidal joint, Hand injuries

## Abstract

Simultaneous dislocations of multiple carpometacarpal (CMC) joints combined with trapezium fractures and trapezoid dislocations at the scaphotrapeziotrapezoidal (STT) joint are extremely rare and often overlooked during initial evaluation, especially in polytrauma settings. Early diagnosis is challenging due to subtle radiographic findings and the potential presence of more life-threatening injuries. While X-rays are useful for the initial evaluation, advanced imaging modalities such as computed tomography (CT) and magnetic resonance imaging (MRI) are crucial for accurately identifying fracture patterns and associated dislocations. Treatment options range from closed reduction and immobilization to surgical fixation, depending on the complexity and stability of the injuries. Prompt recognition and appropriate intervention are essential for optimal functional recovery.

## Highlights

• Simultaneous CMC and STT dislocation with trapezium fracture is extremely rare.
• Carpal fracture-dislocations are often missed on initial assessment.
• CT and MRI help diagnose and visualise carpal fracture-dislocation.
• CMC and STT joints were stabilised using closed reduction and K-wire fixation.

## Introduction

Carpal fractures and dislocations other than the scaphoid are relatively rare injuries, accounting for 1.1% of all fractures, and are often associated with high-energy injuries such as a motor vehicle collision. Among all the carpal fractures, scaphoid and triquetrum fractures are relatively frequent and injuries such as multiple CMC joint dislocation, trapezium fracture and trapezoid dislocation are uncommon [1], [2]. We have experienced concomitant CMC dislocations, trapezium fracture and trapezoid dislocation, and hereby report it.

## Case report

**Fig. 1:**
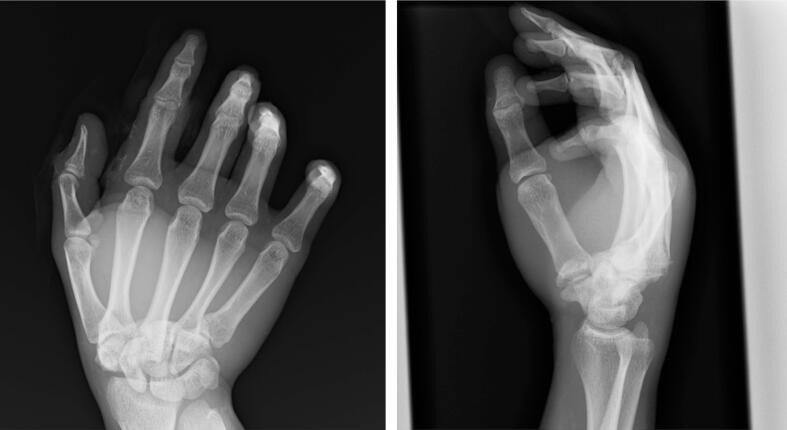
X-rays show dorsal dislocation of the third and fourth CMC joints and loss of the normal articulation of the STT joint.

**Fig. 2:**
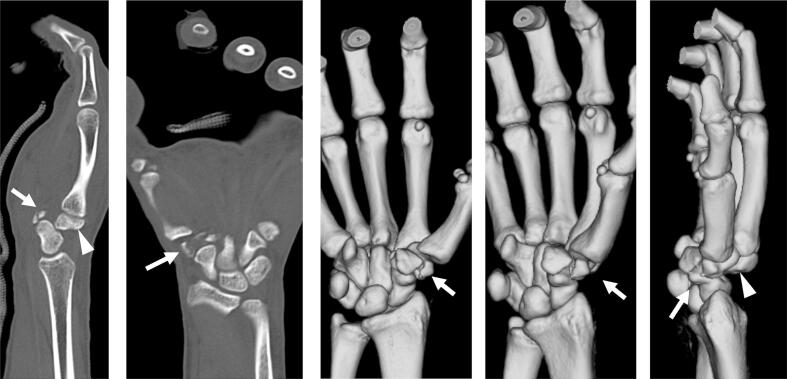
Computed tomography scan demonstrates trapezium fracture (white arrows) and dislocation of the trapezoid (white arrowheads).

**Fig. 3:**
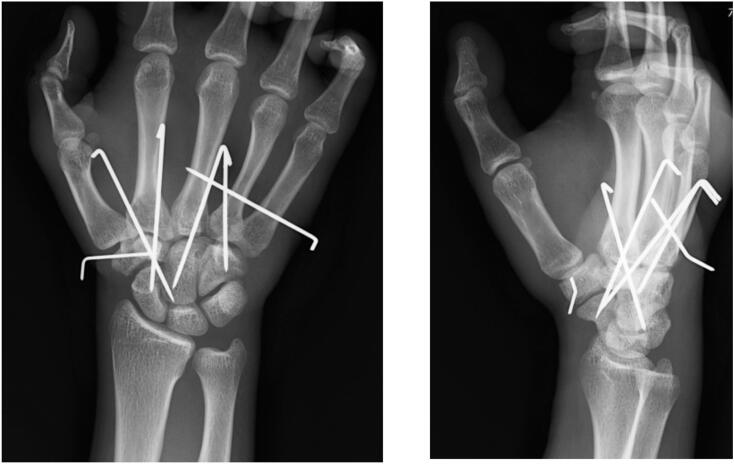
Postoperative X-rays after closed reduction and percutaneous pinning. The STT joint dislocation was reduced with longitudinal traction and transfixed with K-wires. CMC joints were stabilised with a K-wire from the fifth to the third metacarpal shafts.

**Fig. 4:**
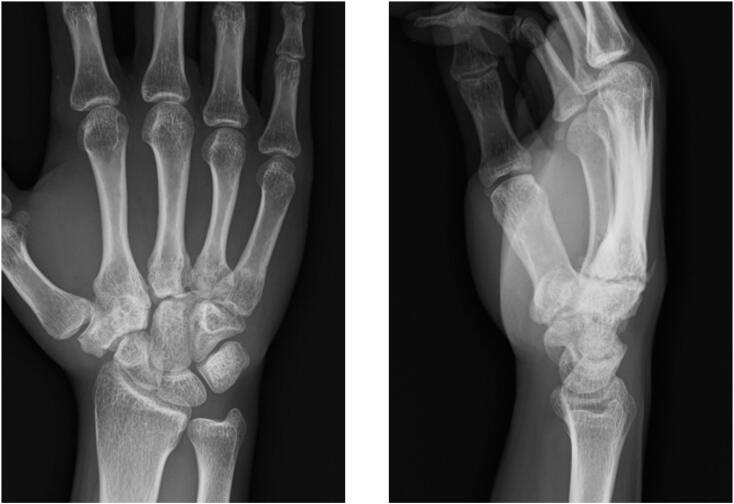
X-rays at the final follow-up, 1 year postoperatively, demonstrate a healed trapezium, and well-aligned STT and CMC joints.

A 22-year-old right-handed male, involved in a frontal motor vehicle collision and brought to our emergency department, complained pain and dysfunction on his right hand. His right hand had been on the steering wheel at the time of the collision. Clinical examination revealed swelling and deformity over the dorsum of the hand. No neurovascular deficit was observed. The initial X-rays showed dorsal dislocations of the third and fourth CMC joints and loss of the normal articulation of the STT joint (Fig. 1). Manual reduction was immediately attempted by applying longitudinal traction to the digits with pressure over the bases of the proximal metacarpals under local anesthesia in the emergency department. Both CMC and STT joints were reduced. However, STT joint remained markedly unstable and difficult to maintain in a reduced position. For further assessment, a CT scan was obtained which revealed trapezium fracture and trapezoid dislocation (Fig. 2). Closed reduction and percutaneous pinning were performed three days after the injury (Fig. 3). STT joint fracture and dislocation were reduced by applying longitudinal traction to the thumb and index finger and transfixed with K-wires. The third and fourth CMC joints were also transfixed followed by transverse fixation from the fifth to the third metacarpal shafts with K-wires. Postoperatively, a splint was applied for 2 weeks and K-wires were progressively removed in 5 weeks. The patient started gentle range of motion (ROM) exercises under the supervision of hand therapists at 1 week. He returned to his former work as a vehicle mechanic at 4 months postoperatively. At the final follow-up at 1 year, the ROM of the wrist extension and flexion was 85° and 82°, which was 94% and 91% of the contralateral side, respectively. The grip strength was 35.6 kg, which was 76% of the contralateral side (Fig. 4). The Mayo wrist score and the quick DASH score were 80 and 0, respectively.

## Discussion

Combined trapezium fracture, trapezoid dislocation and finger CMC joint dislocations are extremely rare and have not been previously described.

Trapezium fractures, with or without other associated injuries, are uncommon and represent only 1 to 5% of all carpal fractures. Walker et al. [3] classified trapezium fractures into five patterns and the fracture pattern in this case was type IV, a two-part fracture involving both the CMC and scaphotrapezial joints. The mechanism of this injury involves either direct dorsoradial impaction or indirect axial loading. The treatment of trapezial body fractures varies from conservative to open reduction and internal fixation.

Major carpal dislocations are known to occur as a result of high-energy and life-threatening injuries including motor vehicle collisions or falls from heights. Trapezoid dislocation at the STT joint is also a rare injury which has been reported as an isolated case or in small case series [4], [5], [6]. Because the trapezoid is firmly anchored in the distal carpal row and attached to its neighboring carpal bones by firm ligaments, significant forces are required to cause a dislocation. The exact mechanism of dorsal dislocation of the trapezoid is unclear but includes a blow to the distal dorsal end of the second metacarpal with the wrist in slight flexion due to weaker ligamentous attachments dorsally and the wedge shape of the trapezoid at the STT articulation [4], [6]. These rare injuries may be overlooked or ignored on initial presentation because of concomitant life-threatening injuries [4], [7]. CT scan may be required to identify injury at the STT joint when radiographs are inconclusive. Options for the treatment of trapezoid dislocation include closed or open reduction with or without K-wire fixation. In our case, the STT joint was successfully reduced but unstable on the initial day, which warranted K-wire fixation.

Carpometacarpal joint dislocations are uncommon injuries, most commonly affecting the ulnar-side (fourth and fifth rays). The radial-side (second and third) CMC joints are well stabilised by interlocked bony and strong ligamentous structures and the third CMC joint acts as a “key-stone” due to its more proximal location than the other CMC joints [8]. Thus, concurrent dislocations of the third and fourth CMC joints are a rare injury. The most common cause of CMC joint dislocation is high-energy trauma such as motor vehicle collisions, falls from heights, or contact sports including boxing [9], [10], [11].

On X-rays, the integrity of three carpal arcs (Guiula's arcs) and the preserved CMC joint spaces are useful to assess CMC joint dislocations. However, the sensitivity of X-rays is still limited. Therefore, CT and magnetic resonance imaging serve as the second-line imaging modalities for the detection of fracture, dislocations and ligamentous injury, when suspected [1], [10], [12].

Treatment options range from conservative treatment, closed reduction with or without percutaneous pinning, open reduction and internal fixation, and CMC joint arthrodesis [1], [10], [11]. Among them, percutaneous pinning is a commonly employed treatment, providing rigid fixation [10]. In our case, the CMC joints were unstable after manual reduction. Therefore, K-wire fixation was performed.

In conclusion, we experienced a case with combined injuries of trapezium fracture, trapezoid dislocation from STT joint, and multiple CMC dislocations. It is essential to suspect such injuries in patients with hand injuries with multiple trauma, and prompt images such as CT and MRI are crucial if suspected. Operative management is usually indicated with closed or open reduction followed by internal fixation.

## CRediT authorship contribution statement

**Yuhei Mogi:** Writing – original draft. **Kinya Nishida:** Supervision, Writing – review & editing. **Maeda Akane:** Writing – review & editing. **Uesugi Kazuhiro:** Writing – review & editing. **Irifune Hideto:** Writing – review & editing. **Che Yong Ho:** Writing – review & editing. **Takahiro Iida:** Writing – review & editing.

## Declaration of competing interest

The authors declare that they have no known competing financial interests or personal relationships that could have appeared to influence the work reported in this paper.
